# Alkaloid profiling and antimicrobial activities of *Papaver glaucum* and *P. decaisnei*

**DOI:** 10.1186/s13104-021-05762-x

**Published:** 2021-09-08

**Authors:** Hawraz Jawdat Jafaar, Ovgu Isbilen, Ender Volkan, Gunay Sariyar

**Affiliations:** 1 Faculty of Pharmacy, Cyprus International University, via Mersin 10, 99258 Nicosia, Northern Cyprus Turkey; 2 Biotechnology Research Center, Cyprus International University, via Mersin 10, 99258 Nicosia, Northern Cyprus Turkey

**Keywords:** *Papaver decaisnei* Hochst. & Steud. Ex Elkan, *Papaver glaucum* Boiss. & Hausskn., Alkaloids, Antimicrobial activity, ^1^H-NMR spectra

## Abstract

**Objective:**

*Papaver decaisnei* Hochst. & Steud. Ex Elkan and *Papaver glaucum* Boiss. & Hausskn. growing wild in Northern Iraq have been historically used for medicinal purposes. In this study, both species were evaluated for their alkaloid content and antimicrobial activities.

**Results:**

Alkaloids were extracted and isolated by preparative thin-layer chromatography (TLC). Identification was carried out by comparing spectral data (UV and ^1^H-NMR) and TLC Rf values with those of authentic samples. Two alkaloids, proapaorphine-type mecambrine and aporphine-type roemerine were isolated from *P. decaisnei*. Two benzylisoquinoline type alkaloids papaverine (major alkaloid) and palaudine as well as aporphine-type *N*-methylasimilobine have been obtained in *P. glaucum*. Both *P. glaucum* and *P. decaisnei* extracts revealed strong antimicrobial activity on *Pseudomonas aeruginosa* ATCC 27853 and *Enterococcus faecalis* ATCC 29212. Collectively these results indicate that *P. glaucum* and *P. decaisnei* are promising sources of alkaloids that could further be investigated for medicinal purposes.

**Supplementary Information:**

The online version contains supplementary material available at 10.1186/s13104-021-05762-x.

## Introduction

*Papaver somniferum* is one of the oldest cultivated medicinal plants and still keeps its importance for the production of its main alkaloids codeine and morphine [1, 2]. Thebaine, another major alkaloid in *P. somniferum,* is utilized as starting material for semisynthetic opioids oxycodone and buprenorphine [2]. Studies on the alkaloid content of different wild Papaver species have revealed many chemotypes, some of which contain thebaine as a major alkaloid along with other medicinally important alkaloids [3]. Alkaloids obtained from Papaver species show biological effects including persuasive narcotic, analgesic, anti-inflammatory, anti-hypertensive, antioxidant, antidepressant, anticancer, antimicrobial, antidiarrheal activities as well as cholesterol and glucose-lowering properties [4, 5].

There are 15 species of genus Papaver growing wild in Iraq grouped under 5 sections namely *Papaver* L., *Carinatae Fedde*, *Rhoeadium*, *Argemonidium* and *Meconidium Bernh* [6]. Section Papaver includes three species *P. somniferum*, *P. decaisnei* and *P. glaucum* [6]. To our knowledge, there is only one report on the alkaloid content of Papaver species from Iraq [3]. Aporphine (liriodenine, roemerine, dehydroroemerine, roemerine *N*-oxides), rhoeadine (rhoeadine, rhoeagenine, glaucamine) and protopine-type (protopine) alkaloids have been shown together with some unidentified alkaloids of aporphine and 1-benzylisoquinoline nature in different samples of Iraqi herbarium materials [3].

The biologically active and medicinally significant nature of plants from genus Papaver have led us to investigate the alkaloids of understudied Papaver species, *P. glaucum* and *P. decaisnei* growing in northern Iraq along with elucidating their medicinal potential via studying their antimicrobial activities *in-vitro*.

## Main text

### Methods

#### Plant material collection

Samples of *P. decaisnei* and *P. glaucum* were collected from Northern Iraq at the flowering stage from Hawraman-Tawela and Rowanduz-Gorge region, Voucher specimens have been identified by Prof. Dr. Neriman Özhatay Chair of Pharmaceutical Botany, Eastern Mediterranean University and Prof. Dr. Mehmet Koyuncu Chair of Pharmaceutical Botany, Cyprus International University and deposited in Cyprus International University Public Herbarium (*P. glaucum* herbarium number: CIUH-126; *P. decaisnei* herbarium number: CIUH-127).

#### Alkaloid extraction and isolation

The dried *P. decaisnei* and *P. glaucum* aerial parts were each percolated with methanol at room temperature and evaporated to obtain syrup-like residue under vacuum. The residue was dissolved in 5% hydrochloric acid (HCl) and extracted with light petroleum. The acidic fraction was made alkaline with 25% ammonia (NH_3_) (Sigma Aldrich, Germany) to pH 7-8 and extracted successively with chloroform (Sigma Aldrich, Germany). Chloroform extracts were combined and dried over anhydrous sodium sulphate (Na_2_SO_4_), filtered and concentrated at low pressure to yield total tertiary alkaloid extracts of *P. decaisnei* and *P. glaucum* [7].

#### Preparative thin layer chromatography (TLC)

Pre-coated Silica Gel Plates: TLC Silica gel 60 F254 Glass Plates 20 × 20 DC Kiesel gel (Merck 1.05715.0001) were used to separate tertiary alkaloids by preparative TLC. UV light (254/366 nm) was used for band detection. Chloroform:Methanol:Ammonia (95:5:0.05) were used as solvent system for *P. glaucum* where Benzene:Ethanol:Ammonia (9:1:0.01) were used for *P. decaisnei.* The bands were scraped and powdered. The alkaloids were then eluted with mixture of chloroform (CHCl_3_) (Sigma Aldrich, Germany) and methanol (MeOH) (Sigma Aldrich, Germany) (9:1) and evaporated to dryness under nitrogen [7].

#### ^1^H-NMR spectrum analysis

The ^1^H Nuclear Magnetic Resonance (NMR) spectra were recorded in deuterochloroform (CDCl_3_) by VARIAN (Agilent) MERCURY 400 MHz (Varian, Palo Alto, CA, USA) spectrometer with 400 MHz proton resonance frequency in Ankara University Central Laboratory.

#### UV spectrum analysis

Shimadzu UV-2401/2501 spectrophotometer was used between 200 and 400 wavelength/nm. Spectra were made with methanol solution for 1 cm quartz cuvettes. Spectra were recorded by adding 50 µl of 0.5 N sodium hydroxide (NaOH) (Sigma Aldrich, Germany) solution to samples. 50 µl of 0.5 N HCl solution was added to sample solutions and the spectrum was recorded again [8].

#### Minimum inhibitory concentration (MIC)

For the MIC assessment, fresh bacterial broth cultures were prepared, standardized to 0.5 McFarland and diluted with sterile PBS to 1 × 10^5^ cfu/ml. The extracts were serially diluted, bacterial culture was added to each tube and incubated at 37 °C for 18 h. The absorbance was measured at 600 nm relative to the blank and the controls. The tests were done three times in triplicates [9].

### Results and discussion

#### Extraction and separation of alkaloids from *P. glaucum* and *P. decaisnei*

The amount of plant material used and the yield of tertiary alkaloid extracts from *P. glaucum* (PG) and *P. decaisnei* (PD) aerial samples was 150 g and 100 g respectively. Extractions of PG and PD lead to 0.24% and 0.11% of extract yield respectively.

#### Isolation and purification of *P. glaucum* and *P. decaisnei* alkaloids by preparative TLC

**Fig. 1:**
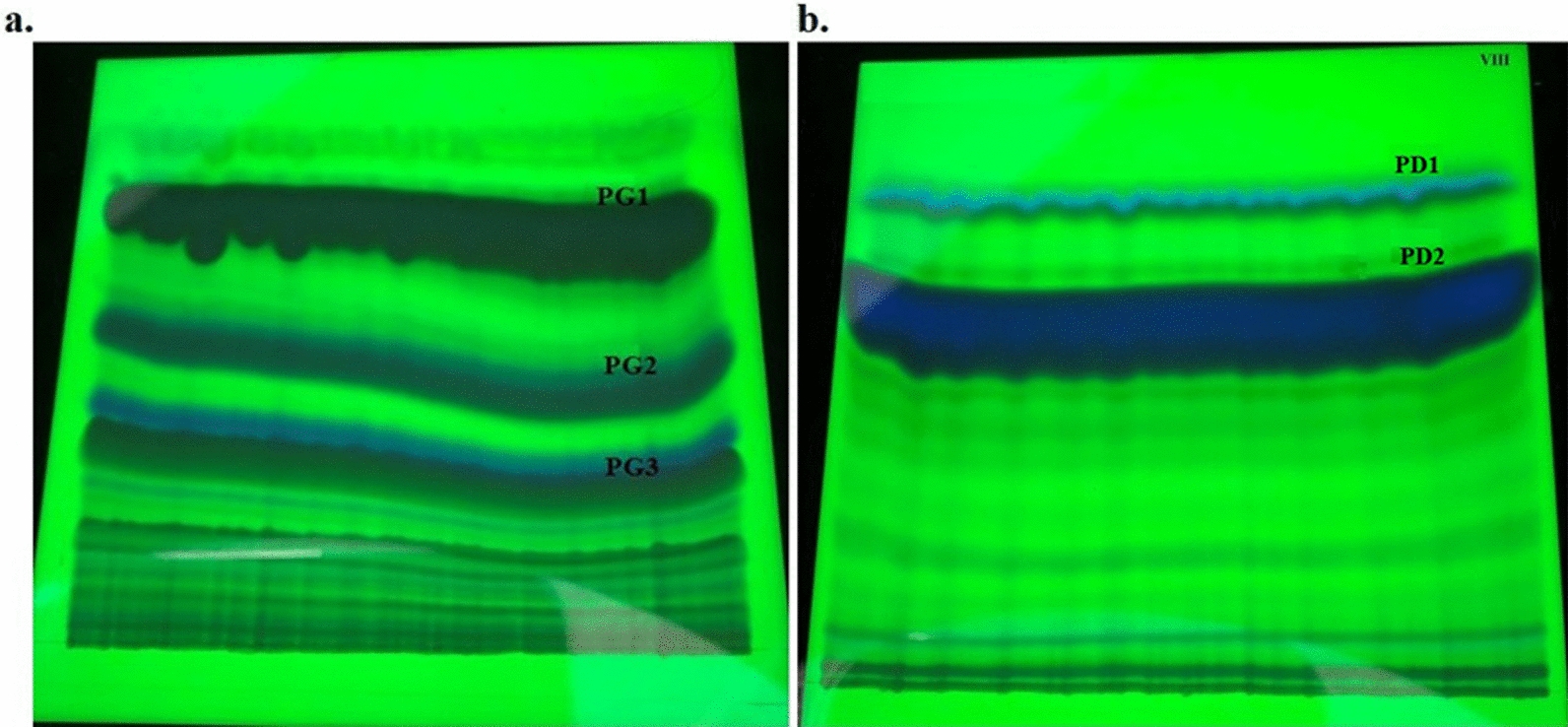
*Papaver glaucum* (PG) (**a**) and *P. decaisnei* (PD) (**b**) alkaloids under UV Light 254/366 nm. TLC plate profile for fractionation of PG using Chloroform:Methanol:Ammonia (95:5:0.05) and PD using Benzene:Ethanol:Ammonia (9:1:0.01), showing specific bands of alkaloids. The three most prominent bands were termed PG1, PG2, PG3 for *Papaver glaucum; *PD1 and PD2 for *P. decaisnei*

Preparative TLC of PG tertiary extract allowed detection of three bands named PG1, PG2 and PG3 (Fig. 1a).

Preparative TLC of PD tertiary extract allowed detection of two prominent bands named PD1 and PD2 (Fig. 1b). The amount of extract obtained from PG and PD was calculated for each fraction (Additional file 2: Table S1).

Rf values obtained from TLC plates of PG and PD were calculated for PG1, PG2 and PG3 in Chloroform:Methanol:Ammonia (95:5:0.05) solvent system, where PD1 and PD2 were calculated in Benzene:Ethanol:Ammonia (9:1:0.01) solvent system (Additional file 3: Table S2).

#### Identification of alkaloids from* P. decaisnei* and *P. glaucum*

**Fig. 2:**
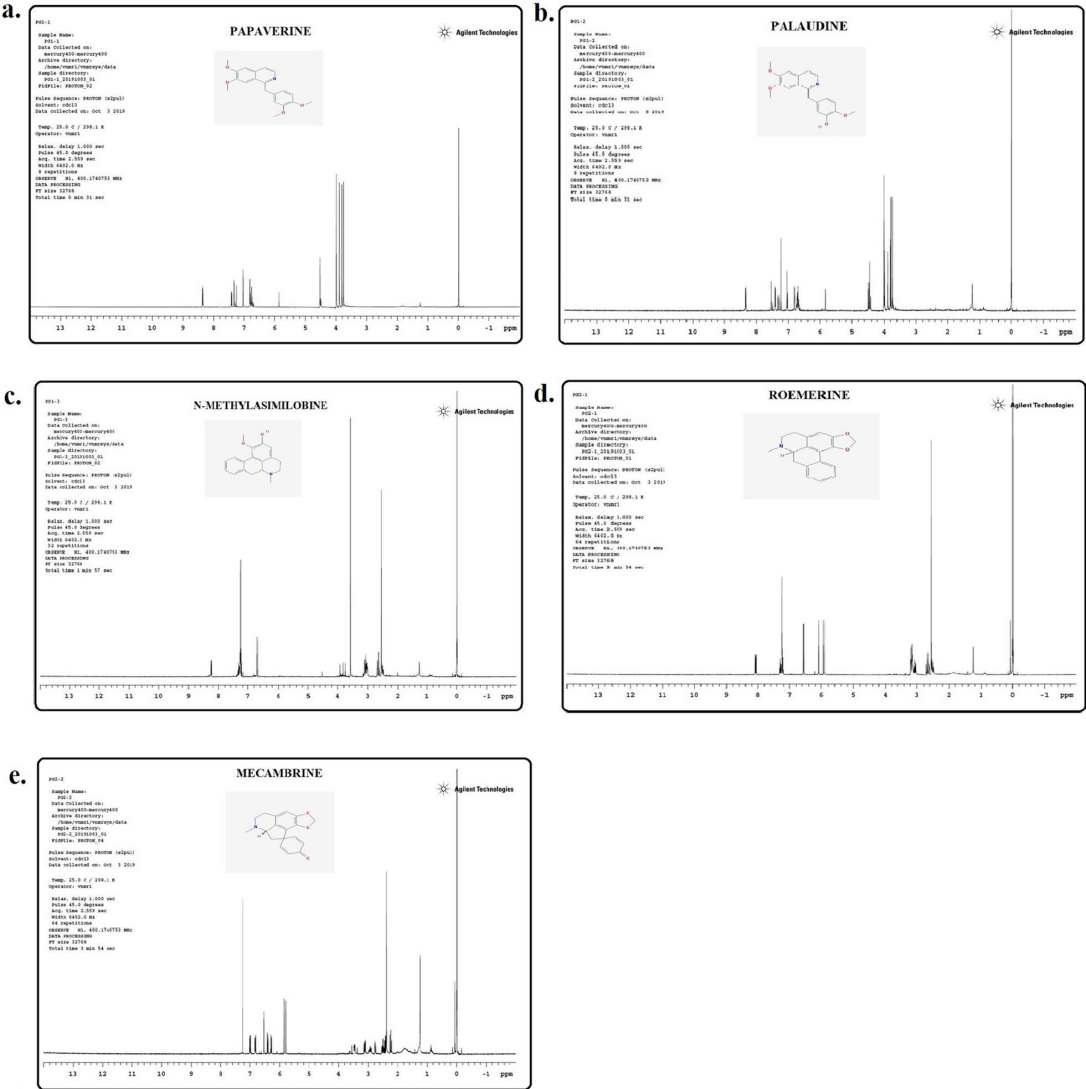
^1^H-NMR spectra of papaverine (**a**), palaudine (**b**), *N*-methylasimilobine (**c**) alkaloids obtained from *Papaver glaucum* and roemerine (**d**) and mecambrine (**e**) alkaloids obtained from *P. decaisnei*

Structure elucidation of alkaloids were carried out based on UV spectroscopic analysis and ^1^H-NMR as well as TLC by direct comparison (except for palaudine) with authentic samples. ^1^H-NMR analysis of PD and PG fractions allowed determination of different alkaloids. Tertiary alkaloids isolated from PG were identified as benzylisoquinoline-types papaverine (PG1: 97 mg; Fig. 2a) and palaudine (PG2: 16 mg; Fig. 2b) as well as aporphine type *N*-methylasimilobine (PG3: 19 mg; Fig. 2c). Two alkaloids belonging to proaporphine-type (PD2: mecambrine, 43 mg; Fig. 2e) and aporphine-type alkaloids (PD1: roemerine, 6 mg; Fig. 2d) were obtained from PD.

While papaverine (PG1) was determined to be the major alkaloid, palaudine (PG2) was revealed as the minor alkaloid. The isolation of palaudine was first reported from *P. somniferum* [10] using HPLC and HPLC–MS studies, followed by PG [11]. Furthermore, the structure of palaudine as a minor alkaloid has been confirmed by ^1^H-NMR spectra and by comparing ^1^H-NMR ppm values with related data [11]. In line with previous studies [10, 11], our UV spectral data further confirm the similarity of palaudine and papaverine (Additional file 1: Fig. 1).

In our study, three signals at 3.75, 3.76 and 4.46 ppm indicated the presence of three OMe at C6, C7 and C4. A methylene signal appears at 4.46 ppm. Presence of four doubles and 3 singlets corresponded to 6 aromatic protons. Methylene signals at 4.46 and at 4.52 ppm in the ^1^H-NMR spectra of palaudine and papaverine respectively confirm their benzylisoquinoline structure (Fig. 2b and Fig. 2a). This is the first report of isolation of a benzylisoquinoline alkaloid papaverine as a major alkaloid in PG. The benzylisoquinoline papaverine and *N*-methylasimilobine have been shown for the first time in this species. However, isolation of *N*-methylasimilobine was not surprising since another aporphine alkaloid liriodenine, structurally similar to *N*-methylasimilobine, was previously shown in PG [3]. In addition to liriodenine, a previous investigation of Iraqi origin PG herbarium specimens reported the identification of roemerine, dehydroromerine, roemerine *N*-oxide, rhoeagenine and glaucamine together with an unidentified aporpine [3].

The alkaloids of PD growing wild in Iraq were studied for the first time in our study. Isolation of total tertiary alkaloids from PD by preparative TLC, yielded two alkaloids, aporphine-type roemerine (PD1) as a minor alkaloid and proaporphine-type mecambrine (PD2) as major alkaloid. These alkaloids were isolated from PD for the first time. ^1^H-NMR spectra of mecambrine and roemerine (Fig. 2e and Fig. 2d) were compared with literature data and TLC Rf values were found to be identical with those of reference samples. Previous investigations of PD have revealed the presence of codeine, coptisine, corytuberine, isorhoeadine, morphine, narcotine, papaverine, papaverrubines, protopine, rhoeadine, roeagenine, thebaine, thebaine methohydroxide, and papaverine as the major alkaloid [12, 13].

#### Antimicrobial activities of extracts from* P. glaucum* and *P. decaisnei*

Various plant extracts and essential oils have been demonstrated to have antibacterial and antifungal activities, impacting even antimicrobial resistant species [14–16]. Multidrug resistant (MDR) bacteria are among the biggest challenges that global health care systems are faced with, as they not only resist classical therapy but they also have improved virulence mechanisms, including increased biofilm formation [17]. This issue requires thorough investigation of novel agents with antimicrobial and/or anti-virulence properties to provide candidates that can be translated as effective treatment approaches [18]. Alkaloids have historically been studied and utilized for their antimicrobial activities [19, 20]. In our study, antibacterial activities of PG and PD alkaloids were investigated using minimum inhibitory concentration (MIC) assessment on both Gram-negative and Gram-positive bacteria. Methanol extracts and tertiary alkaloids obtained from PG and PD showed strong antimicrobial effect on Gram-positive *Enterococcus faecalis* ATCC 29212 and Gram-negative *Pseudomonas aeruginosa* ATCC 27853 (Table 1). While all tested methanol extracts exhibited antimicrobial effect, total tertiary alkaloids from both species demonstrated the most potent antimicrobial activity (Table 1). Particularly PDTA (*P. decaisnei* total tertiary alkaloids) exerted antimicrobial effect on both of the tested bacteria at 27.5 μg/ml, the lowest concentration of all tested alkaloids. *P. glaucum* tertiary alkaloids (PGTA) demonstrated antimicrobial activity on both tested microorganisms starting at 47 μg/ml (Table 1). Methanolic extracts from both PG and PD exhibited antimicrobial effect, however with reduced potency, when compared with isolated total tertiary alkaloids (Table 1). The MIC values for methanol extracts were 2–four fold higher for PG and about seven fold higher for PD when compared with their total tertiary alkaloids, highlighting total tertiary alkaloids’ effectivity (Table 1). PGME (*P. glaucum* methanol extracts) exhibited a fold increase in antimicrobial activity towards the Gram-negative *P. aeruginosa* (90 μg/ml) compared to the Gram-positive *E. faecalis* (180 μg/ml). Collectively, our results reveal the strong antimicrobial activity of PG and PD extracts, especially highlighting the antibacterial effectivity of their total tertiary alkaloids.

**Table 1:** Minimum inhibitory concentration (MIC, μg/ml) of methanol and total tertiary alkaloid extracts from PG and PD

PG and PD extracts	*Enterococcus faecalis* ATCC 29212	*Pseudomonas aeruginosa* ATCC 27853
PGME	180 μg/ml	90 μg/ml
PDME	200 μg/ml	200 μg/ml
PGTA	47 μg/ml	47 μg/ml
PDTA	27.5 μg/ml	27.5 μg/ml
Ampicillin	3.12 μg/ml	1.56 μg/ml

PGME: *P. glaucum* ethanol extract; PDME: *P. decaisnei* methanol extract; PGTA: *P. glaucum* total tertiary alkaloids; PDTA: *P. decaisnei* total tertiary alkaloids

In addition to their antibacterial activity [21], alkaloids have antibiotic enhancing and antivirulence activities where they served as scaffolds for important antibacterial drugs like metronidazole and quinolones [22]. Alkaloids mostly exhibit antibacterial activity through depolarizing the cell wall, intercalating into bacterial DNA and inhibiting mRNA transcription [23, 24]. Anti-virulence properties associated with alkaloids include preventing biofilm formation via interfering with regulation of virulence genes as well as impacting quorum sensing and expression or function of sortases, adhesins and various secretion systems [25].

While mecambrine and roemerine may synergistically be contributing to the antimicrobial activity observed with PD alkaloids, strong antimicrobial effect is likely roemerine mediated. Tsai and co-workers have previously shown antimicrobial effects of roemerine on *Bacillus cereus*, *Micrococcus* sp, and *Staphylococcus aureus* [26]. Roemerine was previously reported to alter *S. aureus* membrane permeability [27].

*N*-methylasimilobine alkaloid whose phenolic character was confirmed by UV spectra taken in 0.5 N NaOH (Additional file 1: Fig. S1) [28], was thought to contribute towards antimicrobial activity against Gram-positive bacteria [29] in addition to having moderate antioxidant capacity [30].

### Conclusion

Overall, our study provides a thorough investigation of the alkaloid contents of two understudied species, *P. glaucum* and *P. decaisnei*. We report, for the first time, the isolation of roemerine and mecambrine from *P. decaisnei* and isolation of papaverine and *N*-methlyasimilobine from *P. glaucum* while further establishing benzylisoquinoline alkaloid papaverine as a major alkaloid in *P. glaucum*. These Papaver species and their extracts were also studied for the first time for their antimicrobial properties where their antimicrobial activities against pathogenic microorganisms, *E. faecalis* and *P. aeruginosa* were elucidated. We reveal the potential of these plant species to further be utilized in antimicrobial, antivirulence or synergistic, antibiotic-enhancing studies.

## Limitations of the study

*Papaver glaucum* and *P. decaisnei* samples were collected from wild in Northern Iraq and their alkaloid content were studied in Cyprus. Limited amount of sample allowed investigation of their alkaloid content on limited number of pathogens.

## Supplementary Information

**Additional file 1: Figure S1.** UV visible spectra of *Papaver glaucum* and *P. decaisnei* alkaloids.

**Additional file 2: Table S1.** Isolated alkaloids from PG and PD extract.

**Additional file 3: Table S2.** Rf Values of Alkaloids Obtained from *P. glaucum* (PG) and *P. decaisnei* (PD) Extracts.

## Data Availability

The datasets supporting the conclusions of this article are available upon request and samples are available in the repository of Cyprus International University herbarium.

## Abbreviations

^1^H-NMR: Hydrogen^1^ Proton nuclear magnetic resonance
ABTS: 2, 2'-Azino-bis (3-ethylbenzothiazoline-6-sulfonic) acid
ATCC: American Type Culture Collection
BALB/c: Albino laboratory-bred strain house mouse
cfu/ml: Colony forming unit per millilitre
CIUH: Cyprus International University Herbarium
CHCl_3_: Chloroform
DNA: Deoxyribonucleic acid
DPPH: 2,2-Diphenyl-1 picrylhydrazyl
HCl: Hydrochloric acid
HPLC: High Performance Liquid Chromatography
HPLC–MS: High Performance Liquid Chromatography–Mass Spectrometry
MeOH: Methanol
MHz: Mega Hertz
MIC: Minimum inhibitory concentration
mRNA: Messenger ribonucleic acid
NaOH: Sodium hydroxide
NaSO: Sodium sulphate
NH_3_: Ammonia
OMe: Methoxy group
PD: *P.* *decaisnei*
PG: *P.* *glaucum*
PDME: *P.**decaisnei* methanol extracts
PDTA: *P.**decaisnei* total tertiary alkaloids
PGME: *P.**glaucum* methanol extracts
PGTA: *P.**glaucum* tertiary alkaloids
Ppm: Parts per million
Rf: Retention factor
SV: Solvent-system
TLC: Thin-layer chromatography
UV: Ultraviolet
