# Supplementary figures and images for: Alkaloid profiling and antimicrobial activities of Papaver glaucum and P. decaisnei
Source: BMC Res Notes. 2021 Sep 8;14:348. doi: 10.1186/s13104-021-05762-x (PMC8424945; doi:10.1186/s13104-021-05762-x)

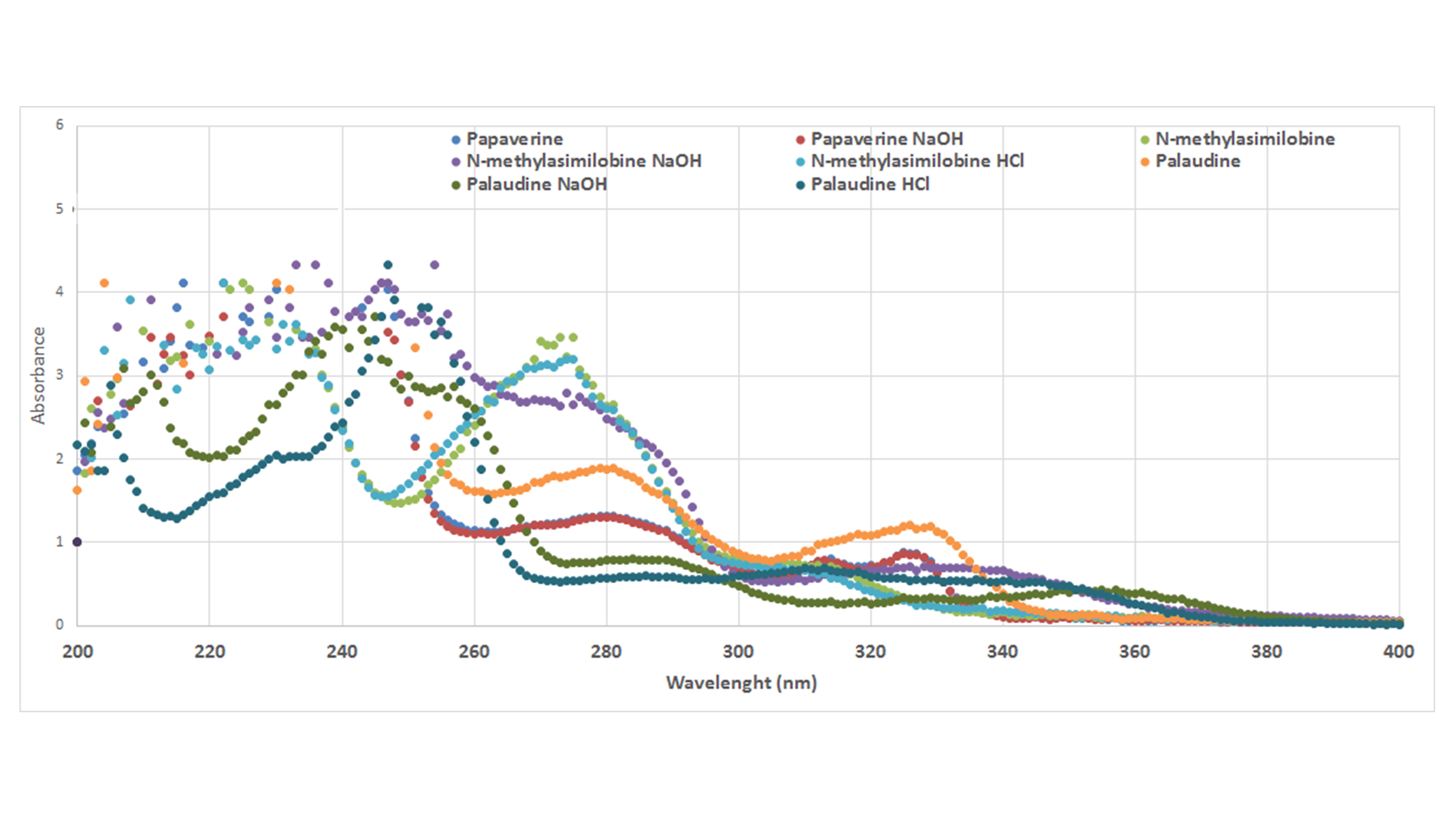

Supplement: Supplementary file 1 — Additional file 1: Figure S1. UV visible spectra of Papaver glaucum and P. decaisnei alkaloids. [file 13104_2021_5762_MOESM1_ESM.jpg]
