# Supplementary material for: Alkaloid profiling and antimicrobial activities of Papaver glaucum and P. decaisnei
Source: BMC Res Notes. 2021 Sep 8;14:348. doi: 10.1186/s13104-021-05762-x (PMC8424945; doi:10.1186/s13104-021-05762-x)
Supplement: Supplementary file 2 — Additional file 2: Table S1. Isolated alkaloids from PG and PD extract. [file 13104_2021_5762_MOESM2_ESM.docx]

**Table S1: Isolated alkaloids from PG and PD extract**

| Alkaloid No | Amount (g) |
| --- | --- |
| PG1 | 0.097 |
| PG2 | 0.016 |
| PG3 | 0.019 |
| PD1 | 0.006 |
| PD2 | 0.043 |
