# Supplementary material for: Alkaloid profiling and antimicrobial activities of Papaver glaucum and P. decaisnei
Source: BMC Res Notes. 2021 Sep 8;14:348. doi: 10.1186/s13104-021-05762-x (PMC8424945; doi:10.1186/s13104-021-05762-x)
Supplement: Supplementary file 3 — Additional file 3: Table S2. Rf Values of Alkaloids Obtained from P. glaucum (PG) and P. decaisnei (PD) Extracts. [file 13104_2021_5762_MOESM3_ESM.docx]

**Table S2: Rf Values of Alkaloids Obtained from *P. glaucum* (PG) and *P. decaisnei* (PD) Extracts**

| Rf Values for Alkaloids | | |
| --- | --- | --- |
|  | **Chloroform: Methanol: Ammonia (95: 5: 0.05)** | **Benzene: Ethanol: Ammonia (9: 1: 0.01)** |
| PG1 | **0.70** | **0.53** |
| PG2 | **0.60** | **0.46** |
| PG3 | **0.35** | **0.26** |
| PD1 | **0.71** | **0.44** |
| PD2 | **0.42** | **0.30** |
